# Is genetic risk of ADHD mediated via dopaminergic mechanism? A study of functional connectivity in ADHD and pharmacologically challenged healthy volunteers with a genetic risk profile

**DOI:** 10.1038/s41398-022-02003-y

**Published:** 2022-06-29

**Authors:** Oliver Grimm, Lara Thomä, Thorsten M. Kranz, Andreas Reif

**Affiliations:** Department of Psychiatry, Psychosomatic Medicine and Psychotherapy, University Hospital Frankfurt, Goethe University, Frankfurt, 60528 Germany

**Keywords:** Neuroscience, ADHD, Pathogenesis, Diagnostic markers

## Abstract

Recent GWAS allow us to calculate polygenic risk scores for ADHD. At the imaging level, resting-state fMRI analyses have given us valuable insights into changes in connectivity patterns in ADHD patients. However, no study has yet attempted to combine these two different levels of investigation. For this endeavor, we used a dopaminergic challenge fMRI study (L-DOPA) in healthy participants who were genotyped for their ADHD, MDD, schizophrenia, and body height polygenic risk score (PRS) and compared results with a study comparing ADHD patients and healthy controls. Our objective was to evaluate how L-DOPA-induced changes of reward-system-related FC are dependent on the individual polygenic risk score. FMRI imaging was used to evaluate resting-state functional connectivity (FC) of targeted subcortical structures in 27 ADHD patients and matched controls. In a second study, we evaluated the effect of ADHD and non-ADHD PRS in a L-DOPA-based pharmaco-fMRI-challenge in 34 healthy volunteers. The functional connectivity between the putamen and parietal lobe was decreased in ADHD patients. In healthy volunteers, the FC between putamen and parietal lobe was lower in ADHD high genetic risk participants. This direction of connectivity was reversed during L-DOPA challenge. Further findings are described for other dopaminergic subcortical structures. The FC between the putamen and the attention network showed the most consistent change in patients as well as in high-risk participants. Our results suggest that FC of the dorsal attention network is altered in adult ADHD as well as in healthy controls with higher genetic risk.

## Introduction

Attention-deficit hyperactivity/disorder (ADHD) is a neurodevelopmental disorder characterized by the onset of developmentally inappropriate levels of impaired inattention, hyperactivity, and impulsivity during childhood. ADHD is one of the most common mental disorders, with a worldwide prevalence estimate of 3–6% and a heritability estimate of more than 75% [1]. ADHD is not restricted to childhood but shows a variable persistence in adulthood. Childhood and adulthood ADHD show a high degree of shared heritability [2] but the factors that determine the trajectory of the disorder are largely unknown. Polygenic risk scores (PRS) for ADHD can be derived from a recent genome-wide association study (GWAS) [3], which provides the opportunity to calculate individual genetic risk. Indeed, this has been successfully performed in a variety of studies for ADHD subtype stratification, as well as course and therapy prediction [4, 5].

ADHD treatment is based on multilevel intervention ranging from social, psychological to pharmacological approaches. Stimulant treatment in both children, as well as adults, demonstrates very high effect sizes [6]. Pharmacological treatments involve one main pharmacodynamical mechanism: increasing dopamine transmission in the synaptic cleft, especially in target brain hubs like the striatum—linked to motivation—and the frontoparietal network—linked to attention [7]. While candidate gene studies mainly concentrated on genes encoding for the dopamine system, these studies were not well replicated, despite the strong therapeutic effects of dopaminergic stimulants [8]. Convincing pathway analyses, that link ADHD PRS to specific biological mechanisms, are as yet lacking.

The main neuroanatomical hubs of the reward system are the dopaminergic nuclei of the midbrain (e.g., ventral tegmental area and the substantia nigra), which send their efferent axons to the whole brain, but especially to the basal ganglia. There, dopamine has an inhibitory function within this circuitry (particularly the globus pallidus). Not only do hypodopaminergic states lead to motor symptoms like Parkinson’s disease but also to inhibition, anhedonia, and depression mediated via the ventral striatum [9]. Resting-state fMRI connectivity can be used to assess the connectivity within the reward system [10].

Studies looking at resting-state functional connectivity (RSFC) with fMRI resting-state concentrated mainly on children, e.g., [11]. This study reported a decreased functional connectivity (FC) of the ventral striatum with the OFC, hippocampus, and anterior prefrontal cortex (PFC) in ADHD. On the contrary, an increase in functional connectivity of OFC with NAcc and anterior cingulate cortex (ACC) was found in a large (*n* = 247 ADHD cases) study in refs. [12, 13] reported an increase in FC between NAcc and the anterior prefrontal cortex in ADHD cases. In a large study, [14]) the authors were not able to find a specific FC alteration in the reward system using independent component analysis in contrast to more top-down-oriented analyses. In general, striatal connectivity patterns are linked to psychopathology. This has been shown in age-related normative growth charts [15]. In this large study in children and young adults, ADHD severity was related to age-advanced connectivity across the insula and to age-delayed connectivity with the nearby inferior frontal gyrus. Therefore, striatal RSFC is a promising target for understanding ADHD-related alterations of the reward system.

**Fig. 1:**
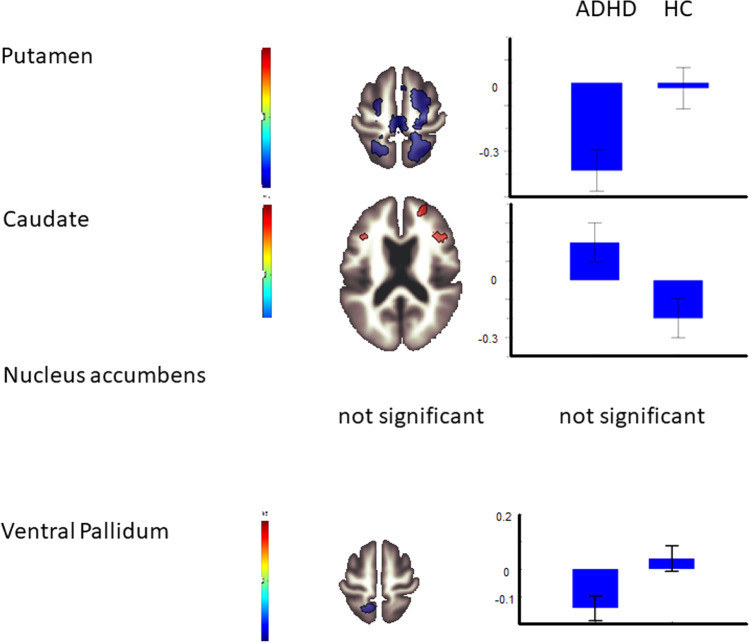
Connectivity in ADHD > HC. The left column gives the name of the seed region-of-interest. The middle column titled shows an exemplary brain slice depicting a significant brain cluster. Color is given in the color bar which codes effect size in red or blue. The column on the right gives mean extracted beta-values for the respective comparison between ADHD and HC to demonstrate absolute values and direction of the effect. ADHD attention-deficit hyperactivity disorder, HC healthy controls.

The reward system is discussed as a common denominator for ADHD and related comorbid disorders [16]. Therefore, it is a promising research line to investigate the influence of dopaminergic motivational circuits in ADHD to better understand the underlying pathophysiologic mechanisms. In line with this proposition, a previous pharmaco-fMRI study demonstrated the influence of L-DOPA on resting-state functional connectivity from dopaminergic subcortical seed nuclei [17]. In our study our interest was twofold: First, we were interested in testing seed ROI functional connectivity in *n* = 27 adult ADHD patients versus *n* = 27 controls to replicate previous L-DOPA-dependent RSFC in ADHD patients (Fig. 1).

We used a neuroanatomically motivated parcellation of the basal ganglia (see Fig. 3, left and middle). Caudate (Ca), putamen (Pu), and nucleus accumbens (NAcc) are important hubs of cognition and motivation in the basal ganglia [18]. In addition, we used the ventral pallidum (VeP), which was recently implicated in reduced pallidal–thalamic pathways associated with deficits in reward-modulated inhibitory control in patients with ADHD [19, 20] sts primarily of rapidly firing GABAergic neurons known to innervate the dopaminergic neurons of the VTA [21]. These areas are coupled e.g., the NAcc innervates the ventral pallidum through GABAergic and GABAergic/glutamatergic projections (indirect versus direct pathway). In sum, bot striatum, as well as VeP, form integral parts of the direct and indirect pathway. Second, we asked how a genetic risk profile (measured by the ADHD PRS) is linked to brain connectivity upon a pharmacological challenge of the dopamine system. While a recent study demonstrated a common dopaminergic gene set for both ADHD and obesity, another disorder linked to a dysregulated dopamine system [22], so far no study investigated the relation between PRS and functioning of the dopaminergic system by probing mechanistically the dopaminergic system. We hypothesized that if PRS impacts on dopaminergic circuitry, we would expect a change of connectivity during a state with higher dopaminergic turnover.

In this study, we concentrated on connectivity from the striatum and related regions. The striatum is not only a major hub for ADHD treatment but also demonstrates an extensive circuitry with the whole cortex. Recent research in humans (fMRI) demonstrated a topographical link between the cortex and striatum [23]. The understanding of disease-specific connectivity changes between the striatum and cortical circuitry gives us new insights into disease mechanisms when considering its relation to polygenic risk give and dopamine. Recent research demonstrated the importance of such a cooperative circuitry between the striatum and cortex for linking stimuli to actions [24]. This dysregulated link between stimuli and action lies at the very basis of ADHD.

## Methods

### Inclusion and exclusion criteria

Inclusion criteria were age (18–50 years). Participants were excluded in case of physical illness or a history of psychiatric disorder. Contraindication to magnetic resonance imaging such as metallic objects in the body lead to exclusion. In the case of the ADHD patients, the diagnosis was confirmed in a specialized outpatient clinic of the university hospital Frankfurt, Goethe University. The diagnosis was based on the ICD-10 criteria and was done by a registered psychiatrist or a psychiatrist in training supervised by an experienced psychiatrist. A semi-structured interview was used as main diagnostic assessment: the diagnostic interview for adult ADHD (DIVA 2.0). This semi-structured interview assesses current and childhood ADHD core symptoms.

### Participants

Participants consisted of two samples, one sample with *n* = 34 healthy volunteers of the pharmaco-fMRI study which were genotyped, and second, *n* = 54 participants of the ADHD versus healthy controls (HC) group. The university clinic’s ethics committee approved the study on August 24, 2016 (ID:256/16).

#### Participants ADHD versus HC study

The participants of both groups were matched according to age and gender. The HC and the ADHD group consisted each of *n* = 27 participants. The mean age for the HC (23.77 a SD 3.04 a) and the ADHD group (24.03 a SD 3.38 a) was not significantly different (*P* = 0.76). Gender distribution was 16 male and 11 female participants for each group. The study was registered in the German study registry on November 11, 2016 under the ID: DRKS00011248.

#### Participants pharmacoMRI study

45 healthy volunteers (average age: 22.81 years, SD: 2.71 years) were included, of whom 22 were male. The average body weight of the subjects included was 72.86 kg (SD: 12.91 kg) with an average height of 1.75 m (SD: 0.11 m). The approval to conduct the study was given by the local ethics commission (Department of Medicine, University Hospital Goethe University Frankfurt am Main) and is subject to the Declaration of Helsinki of the “World Medical Association: Ethical Principles for Medical Research Involving Human Subjects” and the “Guidelines for Good Clinical Practices (GCP)”). The study was registered in the German study registry on November 11, 2016 under the ID: DRKS00011209.

### Experimental procedure: drug application

The measurements were performed at the Brain Imaging Center (BIC) in Frankfurt am Main as a placebo-controlled, double-blind, 3-stage cross-over study. Participants received placebo, L-DOPA or amisulpride in a cross-over study design. In this paper, we report only on the L-DOPA and the placebo sessions because of ambiguous results and interpretation with amisulpride [17].

Participants received a placebo or 125 mg levodopa ~75 min before the start of the resting-state measurement. The session with 200 mg mg amisulpride was not included in our analysis (cf Grimm et al. 2020). Participants received each of the medications exactly once in a counterbalanced order.

### Polygenic risk score

Genotype data were generated using the PsychChip array (15048346 B) with HumanCore, Human Exome, and custom psych content. Normalized intensity values were obtained using Illumina’s. GenomeStudio v2010.3 with the calling algorithm/genotyping module version 1.8.4. Individuals with a call rate >95% were included in the final sample.

PRSs were computed for participants of the pharmaco-fMRI experiment with available blood samples (*n* = 34 out of 45) with available GWAS data using PRSice2 software (http://www.prsice.info/). The reference dataset for ADHD estimation of SNP-wise ADHD risk was based on a recent GWAS [3]. In addition, we calculated PRS based on GWAS for schizophrenia [25] and MDD (as both have been discussed as disorders of dopaminergic dysregulation) to look for specificity in another dopaminergically driven psychiatric disorder. As non-psychiatric control PRS, we choose a recent height GWAS [26]. An *r*^2^ ≥ 0.1 (250-kb window) was used for clumping to remove SNPs in linkage disequilibrium. We controlled for population stratification by including four principal components as covariates for population stratification, regressed them out of the PRS and used the residual for the calculation of linear regression models.

### MRI measurement

The data acquisition was done with a 3 Tesla full-body MR scanner (Siemens Magnetom Trio syngo MR A35, Brain Imaging Center, Frankfurt am Main) and an eight-channel head coil. A T1-weighted sequence (MPRAGE) with a duration of 4:28 minutes was measured and afterward a gradient echo sequence for the functional imaging data was performed, which lasted 8:01 minutes. The sequence information for the MPRAGE sequence are as follows: repetition time (TR) = 1900 ms, echo time (TE) = 3.04 ms, TI = 900 ms, flip angle = 9, FoV (field of view) = 256 × 256 mm, voxel size = 1 × 1 × 1 mm. And for the EPI sequence: repetition time (TR) = 1800 ms, echo time (TE) = 30 ms, flip angel = 90, FoV (field of view) = 192 × 192 mm, m, 28 layers with 4 mm, voxel size = 3 × 3 × 4 mm. Details were described in ref. [27]. Foam pads were used to minimize the head movements of the test persons.

### FMRI data processing

Images were realigned, slice-time corrected, spatially normalized to standard stereotactic space (Montreal Neurological Institute [MNI] template), resampled to 3-mm isotropic voxels, and smoothed with 8 mm full-width at half-maximum Gaussian kernel. A band-pass filtering was used in the frequency band frequency bands to 0.01–0.1 Hz to get rid of non-neural signals. Further noise correction was done by regressing out motion parameters and the 1st order derivative. Signal from the cerebrospinal fluid and white matter was regressed out with the aCompCor-strategy. For seed-voxel connectivity, we used the region-of-interest (ROI) masks from the high-resolution probabilistic in vivo atlas of human subcortical brain nuclei (CIT168) [28] to model connectivity changes from dopaminergic midbrain and subcortical nuclei to the brain. This atlas was constructed out of 168 adults for better delineation of dopaminergic structures. From the available ROIs, we choose the following four due to a priori considerations as seed regions: caudate nucleus, putamen, nucleus accumbens, ventral pallidum.

### Statistical analysis

A power analysis was done with GPower 3.1 [29] to estimate sample sizes needed for reasonable effect sizes. We calculated (with GPower 3.1) effect size of *d* = 0.7 for an alpha of 0.05 with a beta power of 0.8 for group comparison of *n* = 26 per group. For the pharmaco-fMRI study, we calculated a sample size needed for an effect of the pharmacological agent (*d* = 0.5) with 27 participants for a dependent test.

A comparison of age among ADHD and HC group was done with an independent *t* test (in SPSS 25). For the analysis. FMRI group-level statistics were calculated in the CONN toolbox V1.7 [30]. For the ADHD versus HC comparison, we calculated an independent *t* test for each of the a priori-defined seed ROIs. For the analysis of the pharmaco-fMRI experiment, we calculated two types of group statistics. First, an estimation of the correlation between RSFC and PRS without pharmacological stimulation (only placebo session) by a linear regression with PRS was calculated and second, a between-session effect (L-DOPA versus placebo) was added to the model.

For all fMRI group statistics, we used a clusterwise p-FDR-correction, with a cluster defining threshold of <0.001.

## Results

### Comparison of seed-voxel connectivity in ADHD patients versus healthy controls

Table 1 depicts the results of the seed region-based connectivity analysis of the comparison between ADHD and controls. A graphical overview is available in Fig. 1. Three seed ROIs gave significant clusters, no effect was found for the seed nucleus accumbens. The putamen demonstrated stronger connectivity in HC compared to ADHD, namely in a large cluster including pre- and postcentral gyrus bilaterally, superior parietal lobule bilaterally, the frontal gyrus bilaterally, the bilateral superior occipital cortex, and the precuneus. The functional connectivity from the putamen to the thalamus was larger in ADHD cases than in HC controls. The caudate nucleus showed an increase in functional connectivity to the right middle and inferior frontal gyrus, a part of the prefrontal cortex, in ADHD cases in comparison to HC. The ventral pallidum showed a decrease in FC to the pre- and postcentral gyrus, the superior parietal lobule, the lat. superior occipital cortex, and the precuneus in ADHD.

**Table 1:** Comparison of Functional connectivity in a priori seed masks to whole-brain significant cluster between ADHD patients and healthy controls.

Seed ROI	Brain region	Cluster size	MNI coordinates (*X Y Z*)	p-FDR	Effect size *T*
Pu	L + R precentral gyrus. L superior parietal, superior frontal gyrus L, frontal gyrus sup. L, lat. sup. occipital cortex L, precuneus, postcentral gyrus L + R, middle frontal gyrus L + R	1251	−20 −54 +56	<0.001	−6.32
	Superior frontal gyrus R, precentral gyrus R, middle frontal gyrus R, postcentral gyrus R, suppl. motor cortex	1064	+18 +00 +56	<0.001	−6.07
	Superior parietal lobe R, lat. sup. Occipital lobe R, precuneus, postcentral gyrus R	821	+16 −56 +52	<0.001	−5.18
	Thalamus	185	−08 −18 +10	0.017	5.31
Ca	Middle frontal gyrus R, inferior frontal gyrus R	164	+40 +22 +20	0.049	4.87
NAc	Not significant				
VeP	Superior parietal lobe L, precuneus, lat. sup. occipital lobe L, postcentral gyrus L, precentral gyrus L	449	−12 −60 +74	<0.001	−5.36

*ROI* region-of-interest, *Pu* putamen, *Ca* caudate, *NAc* nucleus accumbens, *VeP* ventral pallidum, *L* left, *R* right.

The table gives results for the two-sided comparison ADHD > HC: ADHD > HC has positive effect sizes, HC > ADHD negative effect sizes. Representation of the significant clusters, their size in voxels, and their localization in MNI space as MNI coordinates in the order *X Y Z*, p-FDR, *T*, and beta-values. The brain region with the largest proportion of each cluster is listed. The threshold was set to 0.001 (uncorrected) at the voxel level and p-FDR = 0.05 at the cluster level.

### Correlation between genetic risk and connectivity (in the placebo condition)

**Fig. 2:**
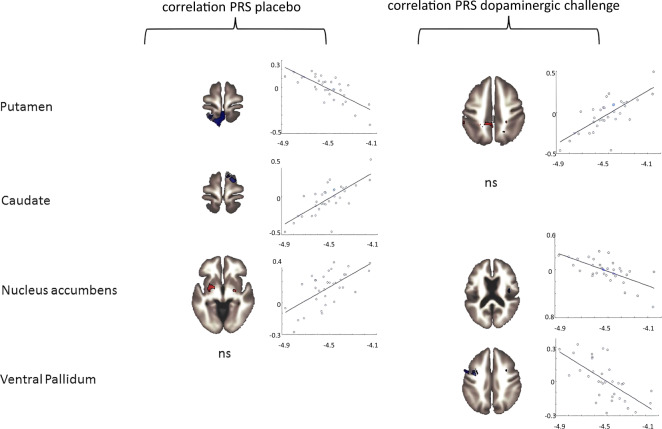
Relation between genetic risk, functional connectivity, and dopaminergic challenge. The left column gives the name of the seed region-of-interest. The column titled “correlation PRS placebo” shows an exemplary slice of a brain with a significant cluster, the scatterplot gives the direction of the effect. The column on the right “correlation PRS dopaminergic challenge” gives the same, slice with significant cluster and scatterplot, for the intrasubject difference L-DOPA > placebo in dependence of the polygenic risk. The scatterplots show the functional connectivity gives as correlation and the difference between L-DOPA and placebo on the *y* axis and the polygenic risk on the *x* axis. PRS polygenic risk, ns not significant.

Table 2 depicts the results of the seed region-based connectivity analysis of the comparison between ADHD and controls. A graphical overview is available in Fig. 2 (left column).

**Table 2:** Correlation of polygenic risk scores (PRS) with functional connectivity.

Seed ROI	Brain region	Cluster size	MNI coordinates (*X Y Z*)	p-FDR	Effect size *T*
Pu	Precuneus, superior parietalis lobe L, postcentral gyrus L, posterior cingulate gyrus	1065	+22 −38 +46	<0.001	−6.79
	Supramarginal gyrus L, angular gyrus	384	−68 −38 +34	<0.001	−6.49
Ca	Orbitofrontal cortex R, temporal pole R, inf. frontal gyrus R	227	+48 +20 −12	0.001	5.93
	Sup. and post. temporal gyrus sup. post.	118	−62 −26 −02	0.02	5.08
	Fusiform gyrus L, lingual gyrus L	111	−20 −86 −12	0,02	5.96
NAC	Putamen, pallidum	144	−30 −04 +00	0.009	4.63
	precuneus	113	−02 −50 +58	0.017	−4.94
VeP	Not significant				

*ROI* region-of-interest, *Pu* putamen, *Ca* caudate.

The table gives results for the correlation of polygenic risk scores (PRS) during the placebo condition. Representation of the significant clusters, their size in voxels, and their localization in MNI space as MNI coordinates in the order *X Y Z*, p-FDR, *T*, and beta-values. The brain region with the largest proportion of each cluster is listed. The threshold was set to 0.001 (uncorrected) at the voxel level and p-FDR = 0.05 at the cluster level.

The putamen in ADHD patients showed a decrease in functional connectivity to a large cluster comprising parts of the precuneus and the superior parietal lobe. While not exclusively centered on the parietal lobe with some parts of the postcentral gyrus, we interpret the main part of the cluster as belonging to the dorsal attention network. In addition, the participants with a higher genetic risk were found to have lower FC to the angular gyrus, another major hub of the dorsal attention network. The FC between caudate and parts of the prefrontal cortex, namely orbitofrontal as well as inferior frontal gyrus showed an increase in FC for participants with higher genetic risk. The FC between the ventral striatum and other parts of the striatum increased for higher genetic risk and decreased for connection to the precuneus. Whole-brain seed-based FC from the ventral pallidum was not significant.

### Correlation between genetic risk and connectivity during the dopaminergic challenge

Table 3 depicts the results of the seed region-based connectivity analysis of the comparison between ADHD and controls. A graphical overview is available in Fig. 2 (right column).

**Table 3:** Correlation of polygenic risk with the dopaminergic challenge.

Seed region	Brain region	Cluster size	MNI coordinates (*X Y Z*)	*P* FDR	Effect size *T*
Pu	Precuneus, Lobulus parietalis sup. L, lat. sup. Occipital Cortex L	218	−06 −56 +72	<0.001	6.21
	superior parietalis lobe L	143	−28 −48 +26	0.006	5.43
Ca	Not significant				
NAC	Lingual gyrus L + R	224	+08 −76 −02	<0.001	−4.98
	Planum temporale L, posterior temporal gyrus L	91	−46 −32 +00	0.031	−5.27
	Central operculum R, Insula R	73	+36 −16 +24	0.043	−4.83
	Lingual gyrus L + R, occipitalis pole R	71	+02 −94 −14	0.043	−4.55
VeP	Precentral gyrus L, frontal medial gyrus L	149	−38 +00 +30	0.004	−4.88

*ROI* region-of-interest, *Pu* putamen, *Ca* caudate, *NAc* nucleus accumbens, *VeP* ventral pallidum, *L* left, *R* right.

Table 3 gives results for the dopaminergic challenge. In dependence of the polygenic risk score, the table gives results for the directed comparison L-DOPA > placebo, therefore positive *T*-values indicate where the FC increased with L-DOPA whereas negative *T*-values indicate where connectivity increased in placebo for participants with a higher genetic risk profile. Representation of the significant clusters, their size in voxels, and their localization in MNI space as MNI coordinates in the order X Y Z, p-FDR and *T*-values are given in the table. The brain region with the largest proportion of each cluster is listed. The threshold was set to 0.001 (uncorrected) at the voxel level and p-FDR = 0.05 at the cluster level.

The putamen demonstrates a pronounced reactivity to L-DOPA. In participants with a high genetic load, the connectivity between putamen, the superior parietal lobe, and the precuneus was increased by L-DOPA. This was not found for the caudate as a seed region. The nucleus accumbens demonstrated a wide loss in connectivity for a range of clusters from right Insula, occipital lobe, and frontal medial gyrus. These areas are part of the salience network and present with a decreased connectivity during L-DOPA-challenge in those patients with a higher genetic risk. The ventral pallidum demonstrated a decrease in FC to the precentral gyrus in those individuals with a higher genetic risk.

For evaluation of the PRS’ specificity, we extracted the functional connectivity for each participant from the putamen–precuneus connectivity during the L-DOPA challenge. We correlated this with the individual PRS for schizophrenia, MDD, and body height. Neither schizophrenia PRS (*P* = 0.23), MDD (*P* = 0.29) nor body height (*P* = 0.29) showed significant correlation with putamen–precuneus connectivity during dopaminergic stimulation, suggesting that this effect is specific for the ADHD PRS.

## Discussion

**Fig. 3:**
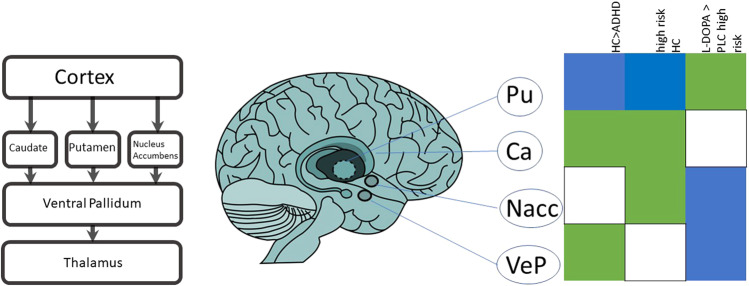
Overview results: direction of connectivity for the specific seed region-of-interest. On the left, a modified overview of the neuronal signal flow from the cortex to the thalamus (and back) gives an impression where in the loop our specific ROI masks are situated. In addition to the well-known motor loop, putamen and caudate are part of an associative and executive loop, whereas the nucleus accumbens is part of the limbic loop. The ventral pallidum is downstream in this loop. The thalamus projects back to the cortex, thus forming a loop (not shown) apart from their motor function. In the middle, a stylized sagittal view gives an impression of neuroanatomical localization. On the right, the matrix gives an abstract overview of the aforementioned results. Green codes a positive direction of the ROI on the left, blue a negative correlation. Each column gives directionality for ADHD > HC, the placebo condition in high-risk participants and in the comparison L-DOPA > placebo in high-risk participants. The main finding. Putamen-derived connectivity shows a directionality switch from the DAN during L-DOPA challenge.

Our study investigated in two separate cohorts and designs, namely ADHD vs HC, as well as a pharmacological challenge in healthy volunteers with PRS profiling, four distinct nodes of the basal ganglia and their consecutive functional connectivity in an fMRI paradigm. This enabled us not only to detect dysregulated connectivity patterns in ADHD patients, but in addition to ask whether similar connectivity patterns are found in healthy participants with higher genetic risk during a dopaminergic challenge. Therefore, we tested for significant changes per se in these samples, second, which areas show significant changes in functional connectivity, and third whether these connectivity changes behave in a uniform way. We discuss in the following section the connectivity changes for each ROI seed in the three conditions patients versus healthy controls (ADHD > HC), placebo genetic risk, and L-DOPA challenge genetic risk.A schematic overview of the result’s directionality is given in Fig. 3 (right).

In ADHD patients, the caudate seed showed stronger connectivity to the prefrontal cortex than in healthy controls. Such hyperconnectivity was previously reported for ventral caudate connectivity to the PFC and is interpreted as neural equivalent of cognitive control over emotional circuits via connections with PFC, ACC, and amygdala. In contrast, dorsal caudate connectivity has been linked to cognitive control over networks dealing with initiation of action and directed movement [14]. However, a higher FC does not fit well in relation with disturbed cognitive control and might be interpreted as a compensatory mechanism. Indeed, in the pharmacological challenge experiment, caudate FC in genetically high-risk individuals was not influenced by a dopaminergic challenge. Therefore, while we did not precisely assess the PRS in ADHD patients but in healthy controls, it is tempting to speculate (coming from our non-ADHD participants in the pharmaco-fMRI-study) that this increase in frontal–caudate FC in ADHD patients is independent of genetic risk; thus pointing to a state and not a trait finding. In a previous study, the ventral caudate did not demonstrate differences between ADHD cases and controls, but a correlation with clinical symptoms, which again is not in accordance with our case and control differences. However, in addition to a slightly different methodology between the studies, the former study was done in adolescents where the reward system might still be subject to maturation. Apart from its role in motor control, the caudate is more linked to frontal-executive pathways compared with more limbic loops of the basal ganglia. This has been demonstrated before with very comparable rs-fMRI-analysis methods [31]. An increase in caudate-prefrontal connectivity in ADHD cases, as well as in those with a higher genetic risk points to a compensatory mechanism, which is independent of dopaminergic influence. In summary, while detecting an interesting increase in fronto-caudate connectivity, we can rule out this connectivity pattern as a genetically mediated dopaminergic main hub of the basal ganglia.

While previous studies have shown that the caudate is more tightly linked to fronto-executive circuitry, it is, therefore, plausible to expect more changes in FC from the seed putamen to the limbic system.

In HC, the putamen showed more FC to the superior parietal lobe and the precuneus in comparison to ADHD cases. The superior parietal lobe is a major hub of the so-called dorsal attention network (DAN) [32] The connectivity between the putamen and the DAN points to the modulation of the transition between the DAN and the default-mode network (DMN) by the salience network (SN). In our study, the putamen (as seed ROI) represents aspects of the SN, especially its prominent dopaminergic innervation. Arguably the most intriguing finding is the that this connection is disturbed in our study, which is in line with previous findings in ADHD [33]. In healthy participants with a higher genetic risk, this connectivity was lowered, which is in accordance with the ADHD HC data. While a recent study in ADHD cases did not detect significant differences from striatal ROIs to the putamen, it indeed found a higher connectivity in those with higher dimensional ADHD scores [14]. However, there are several technical differences to our study, which make direct comparison difficult e.g., lower magnetic scanner strength, different preprocessing, nonparametric statistics etc.

The nucleus accumbens showed a significant increase of connectivity with a higher genetic ADHD risk to the putamen, pallidum and the precuneus. When probed by L-DOPA, this relation was inverted, pointing to a genetically mediated dopaminergic mechanism. Interestingly, this mechanism has no simple correspondence in our ADHD sample, where we could not detect significant NAcc-connectivity differences between HC and ADHD patients. Indeed, several studies in children and adolescents point to enhanced striatal connectivity in NAcc-related connectivity [34, 35]. While this is reflected in the increase of FC with higher genetic risk, it is missing in our adult ADHD sample. This might point to a transition in a genetically-based dopaminergic mechanism of NAcc-connectivity during the step from adolescence to adulthood. However, this needs to be verified in longitudinal cohort studies, which span this life period.

The ventral pallidum is of special interest, as a recent study demonstrated that the nucleus accumbens’ dopamine-D2-receptors increase motivation by decreasing inhibitory transmission to the ventral pallidum [36]. This suggests an important role of the VeP in ADHD-relevant behavior-motivating circuitry in the basal ganglia. While our data did indeed find a significant FC of the VeP in ADHD, the FC to the precentral gyrus and other prefrontal areas were decreased, thus pointing to a circuitry not belonging to the limbic or associative basal ganglia loop. While the VeP showed no change in connectivity depending on the ADHD genetic risk, it nevertheless was sensitive to a dopaminergic challenge: During L-DOPA-stimulation the FC between VeP and the precentral gyrus was reduced. While this points to a lesser efficiency with increased dopaminergic neurotransmission (e.g., curvilinear dependency, cf [17], our seed VeP did not elicit a significant cluster in parts of the limbic cortex as predicted by models pointing to its important role in the reward circuitry. This might stem from technical limits of our FC analysis, leading to more motor-specific parts of the pallidum being observed in our analysis. This is nevertheless of interest, as ADHD has been linked to downregulated connectivity between pallidum and motor cortex in children [37].

The punchline in our study is the dopaminergic challenge: when people with a higher genetic risk (high PRS) receive L-DOPA, increasing their dopamine levels, the functional connectivity increases as well during the dopaminergic challenge. This suggests that people with a genetic predisposition to ADHD have a connectivity fingerprint which (i) resembles ADHD in the DAN and (ii) which is strongly modulated by an acute dopaminergic challenge. This can be interpreted as the basis of psychopharmacological treatment. In participants with a higher genetic risk, L-DOPA leads to an increase in functional connectivity in the dorsal attention network but a decrease in the accumbens-related connectivity in the salience network. When interpreted with ADHD in mind, this demonstrates that dopamine is able to shift connectivity patterns from a “hot” subcortical salience network to a “cold” cortical attention network. Such a distinction between “hot” and “cold” circuitry has been proposed to underly ADHD-related network pathology [38].

A recent meta-analysis of more than 700 patients with ADHD documented decreased connectivity between the FPN and the dorsal attention network, as well as the somatosensory network. The latter is comparable to our description of a VeP-network. Furthermore, this meta-analysis documented a hyperconnectivity between the FPN and the affective networks and/or the salience network. The latter corresponds in our approach to the connectivity of the nucleus accumbens as well as the DMN [39]. According to this model, ADHD results in DMN interference with task-positive networks. Our findings suggest that the genetic reactivity of dopaminergic circuits conveys a counter-responsiveness of affective networks compared to networks of the FPN and DAN. In particular, the FPN/DAN plays a central role in the flexible use of cognitive control. Dopamine modulates the (hyper-)connectivity between the FPN and affective network, which can lead to increased interference by emotional lability on cognitive processes. In follow-up studies, therefore, the clinical correlate should also be looked at particularly in the area of emotional reactivity [16].

We tested whether the increased reacticity to the L-DOPA challenge was specific for the ADHD PRS by calculating the correlation between FC and a schizophrenia PRS, a MDD PRS and as non-psychiatric control a body height PRS. As these non-ADHD PRS were not significant, we conclude that the effect is a specific feature of the ADHD PRS.

A limitation of our study is the small sample of our explorative genetic analysis in the pharmaco-fMRI study. However, previous power analyses were made for single SNPs or haplotypes, and it is not yet clear whether the sensitivity for polygenic risk scores is better. In addition, it is possible that a pharmacological challenge provides a strong lever in terms of effect size. However, implicit in such a logistically demanding strategy (challenge fMRI in healthy volunteers) are low sample sizes. While such an approach has its conceptual appeal, it should be replicated in other (larger) samples. As other pharmaco-FMRI studies are available [40], we urge to genotype their participants. So far, we would like to point the reader to the well-known problem of low sample sizes [41] and present our study as exploratory demonstration of the link between clinically driven PRS and fMRI-based pharmaco-challenge. However, most pharmaco-fMRI samples are done in healthy participants. Our approach suggests a future study of genetic background (like PRS) during a pharmaco-fMRI study with a dopaminergic challenge in ADHD patients might solve several open questions.

Another limitation of our pharmaco-study is the lack of receptor specificity in L-DOPA. L-DOPA has some sedating effects and is not specific for D1- or D2 receptors. Future studies should investigate the effect of PRS scores on D1- versus D2-receptor signaling pathways, as these have been discussed as having opposing effects [42].

While we discuss our findings in the context of ADHD and its genetically constraint dopaminergic functioning, we must underline an important missing aspect: ADHD is a developmental disorder and the dopaminergic system undergoes changes from childhood, adolescence to adulthood which were beyond the scope of our study. This is exemplified by findings looking at U-shaped effects of striatal connectivity of resting-state-fMRI from childhood to adulthood [15]. It might be possible that the reactivity in pharmaco-fMRI-challenge studies varies depending on age. Nevertheless, the genetic basis might be the same, as the genetic correlation of polygenic risk scores from childhood to adulthood is high [2].

A main advantage in comparison to large-scale studies combining genetics, brain imaging and clinical data, is that our analysis does not rest on purely correlational measures but is based on causal experimental challenge of the dopaminergic system. A drawback of such an approach with its requirements on regulatory and organizational aspects, is its small sample size, which might nevertheless be countered by a larger effect size. Large-scale studies should try to replicate our dopamine- and PRS-specific results in the future.

How does dopamine mediate changes in connectivity? It is plausible that the PRS capture to some degree reactivity of the dopaminergic system: first, a recent study demonstrated an enrichment of dopaminergic gene ontologies in ADHD patients [22], and second, non-dopaminergic genes contributing to the PRS might indeed influence the development of the dopaminergic system [43].

Third, previous neurophysiological experiments point to the modulation of glutamatergic neurotransmission by dopamine [44, 45]. This implies that PRS do not have to be necessarily driven by dopaminergic genes, but by more downstream glutamatergic neurotransmission. An example is PICK1, which provides a link between dopamine and glutamatergic neurotransmission and has been implicated as rare variant in an extended ADHD pedigree [46]. While our study demonstrates the impact of a dopaminergic challenge dependent on the genetic risk profile, the exact nature of these genes that drive the responsivity to dopamine have to be elucidated in future studies.

In sum, our study replicated previous findings revealing a disturbed dorsal attention network in ADHD and in healthy participants dependent on the individual genetic ADHD risk. The DAN was sensitive to a dopaminergic challenge, and this was dependent on the individual genetic risk. Further striatal connectivity networks demonstrated a dependence of their reactivity to the dopaminergic challenge on the genetic risk, especially in accumbens-occipital FC. This indicates that the switch between a “hot” and a ”cold” network in ADHD [38] is mediated by dopamine in dependence on an individual’s genetic risk. This underscores that PRS might not only tag clinical phenomena but can be linked to neurobiologically plausible mechanisms. Future studies should study the responsivity of ADHD to dopaminergic medication, as well as examine the dependence of the connectivity’s response to dopaminergic provocation on the individual genetic risk. The development of the dopaminergic system in dependence of the individual genetic risk might further elucidate ADHD pathophysiology.
